# Semitransparent Perovskite Solar Cells with > 13% Efficiency and 27% Transperancy Using Plasmonic Au Nanorods

**DOI:** 10.1021/acsami.1c22748

**Published:** 2022-02-24

**Authors:** Stener Lie, Annalisa Bruno, Lydia Helena Wong, Lioz Etgar

**Affiliations:** † Singapore-HUJ Alliance for Research and Enterprise (SHARE), Nanomaterials for Energy and Energy-Water Nexus (NEW), Campus for Research Excellence and Technological Enterprise (CREATE), Singapore 138602, Singapore; ‡ School of Material Science and Engineering, Nanyang Technological University, Singapore 639798, Singapore; § Energy Research Institute, Nanyang Technological University, Singapore 637141, Singapore; ∥ Institute of Chemistry, Casali Center for Applied Chemistry, The Hebrew University of Jerusalem, Jerusalem 91904, Israel

**Keywords:** semitransparent solar cell, perovskite, plasmon, Au nanorods, photovoltaic cells, interface engineering

## Abstract

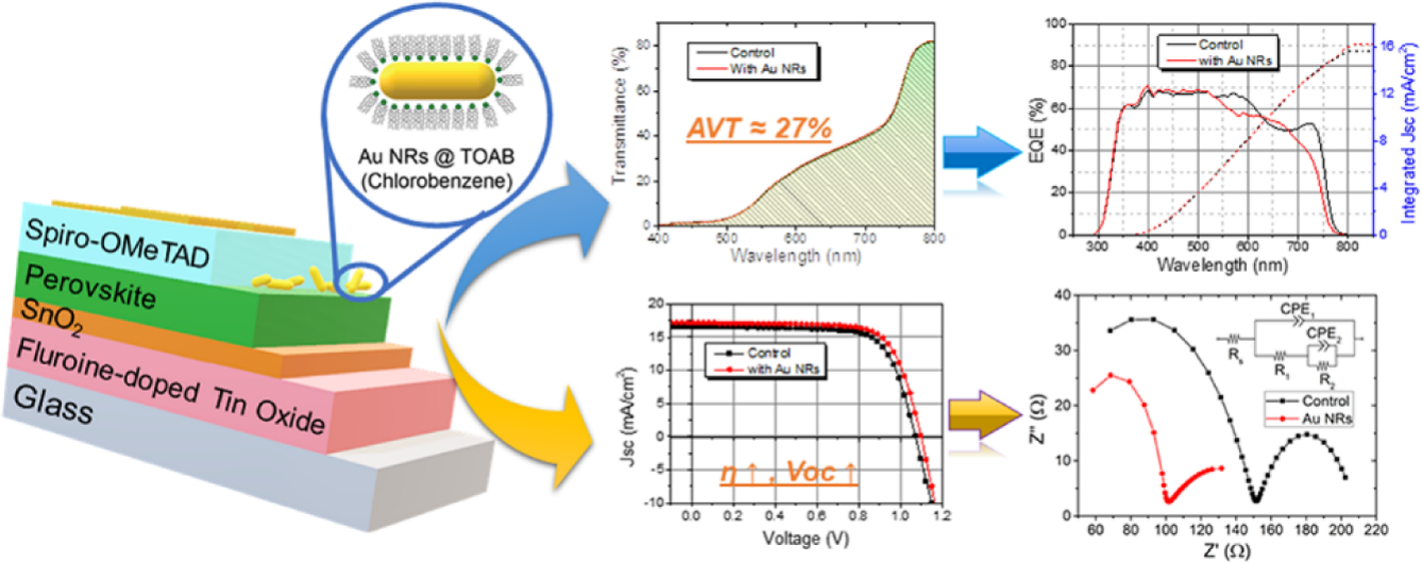

Semitransparent hybrid perovskites open up applications in windows and building-integrated photovoltaics. One way to achieve semitransparency is by thinning the perovskite film, which has several benefits such as cost efficiency and reduction of lead. However, this will result in a reduced light absorbance; therefore, to compromise this loss, it is possible to incorporate plasmonic metal nanostructures, which can trap incident light and locally amplify the electromagnetic field around the resonance peaks. Here, Au nanorods (NRs), which are not detrimental for the perovskite and whose resonance peak overlaps with the perovskite band gap, are deposited on top of a thin (∼200 nm) semitransparent perovskite film. These semitransparent perovskite solar cells with 27% average visible transparency show enhancement in the open-circuit voltage (*V*_oc_) and fill factor, demonstrating 13.7% efficiency (improved by ∼6% compared to reference cells). Space-charge limited current, electrochemical impedance spectroscopy (EIS), and Mott–Schottky analyses shed more light on the trap density, nonradiative recombination, and defect density in these Au NR post-treated semitransparent perovskite solar cells. Furthermore, Au NR implementation enhances the stability of the solar cell under ambient conditions. These findings show the ability to compensate for the light harvesting of semitransparent perovskites using the plasmonic effect.

## Introduction

In order to overcome the increasing energy demand and mitigate climate changes, there is a growing interest in photovoltaic (PV) solar cells, as an alternative energy source. The reliance on silicon as the solar cell technology to fulfill this is impractical; there is a need to supplement silicon especially in specific or niche areas. The perovskite solar cell (PSC) is one of the promising PV technologies, which was developed in recent years due to its low cost, ease of preparation, and interesting and exceptional properties, such as a high optical absorption coefficient, adjustable band gap, and long carrier diffusion length.^1^ In terms of PV performance, the latest power conversion efficiencies (PCEs) of PSCs have exceeded 25%.^2^ Due to their attractive properties, PSCs have the potential to achieve high PV performance as semitransparent solar cells. Semitransparent PSCs with average visible transparency (AVT) between 20 and 30% can reach today PCEs of 8–12%.^3^ One of the main challenges is to maintain high efficiency and high AVT at the same time. For semitransparent PSCs to be a viable technology, their transparency should be controllable, but at the same time, their efficiency should be maximized with each transparency. Although higher transparency is desired for window applications, a small gain in transparency is usually compromised by a larger loss in efficiency; thus, a novel strategy or treatment might be required to gain the best of both sides. There are several options to fabricate semitransparent PSCs, such as a thin perovskite layer, having partial coverage of the perovskite (leaving empty areas on the electron transport layer), or tuning the band gap (let certain wavelength pass through).^4^ Thinning the perovskite layer is the most common method in achieving the semitransparency, which can be done by reducing the concentration. Decreasing the perovskite film thickness is also beneficial in the reduction of the lead amount in the perovskite film. On the other hand, decreasing the thickness will reduce the device performance; thus, there is a need to maintain the light absorption capability of the thin perovskite film as much as possible.

One strategy to do so is by introducing metallic plasmonic particles to enhance the energy conversion efficiency while keeping a thin layer of the light harvester.^5^ This strategy provides unique light gathering and trapping functions, which enhance the solar energy to electricity conversion. This effect comes from localized surface plasmon resonance (LSPR), the collective oscillation of free electrons on the surface of metal nanostructures. This resonance occurs at the metal surface or metal–dielectric interface after electromagnetic radiation.^6,7^ These metal nanostructures are able to capture light radiation at a certain wavelength, and their resonance profile is also customizable depending on the shape, size, and type of metal being used and environment. Plasmonic nanoparticles have proven to boost the stability and PCEs of silicon and organic solar cells.^8^ The use of plasmonic particles in PSCs has shown enhancement in the PV performance by placing them at various locations such as inside the mesoporous TiO_2_ layer,^9^ embedded in the perovskite layer,^10^ or in the hole transporting layer (HTL).^11^ Few studies have investigated the effect of plasmonic particles on the interface between the perovskite and the HTL,^12^ which was found to be a promising strategy to enhance the PV performance. The benefit of plasmonics in a solar cell can be achieved through two main mechanisms.^13^ First, the plasmonic particles act as scattering elements that trap and propagate light, which increases its effective optical length and probability of being absorbed by the solar cell, called the far-field scattering effect. The second is by acting as near-field subwavelength antennas, which allow coupling of the plasmonic particles with the semiconductor, amplifying the electromagnetic field, and enlarging its effective absorption cross-section. This process can also produce hot carriers (above the band gap of photogenerated carriers), which are collected and transferred by plasmonic particles and may demonstrate an increase in the voltage of the solar cell.^14^

The most recognized materials in plasmonics are noble metals such as gold (Au) and silver (Ag) due to their relatively high free-electron densities, ability to resonate within the solar spectrum, lossless capability, and stability.^15^ However, the concerns over the reactivity of silver with the halides in the perovskite make Au the preferred option.^16^ Au is an interesting noble metal due to its ability to exhibit single or multiple LSPR characteristics depending on the size and shape.^17^ In particular, Au nanorods (Au NRs) are able to produce strong resonance peaks near the band gap of the perovskite, which can be adjusted depending on their size.^18^ The longer wavelength absorption is commonly compensated in a very thin perovskite film; thus, having plasmonic resonance around these wavelengths should help reduce it. In addition, the carrier transfer mechanism such as plasmon-induced resonance energy transfer (PIRET) should also occur when the plasmonic resonance peak is overlapping with the band gap of the semiconductor.^19^ The synthesis of Au NRs includes capping using protection layers to stabilize Au from agglomeration. Polymer ligands such as cetrimonium bromide (CTAB),^20^ cetrimonium chloride,^21^ or tetraoctylammonium bromide (TOAB)^22^ are usually used in the seed-based formation of Au NRs. The ligands need to be compatible with the absorber and solvent of choice to deposit the Au NRs. Some ligands which are based on polymers are also found to be beneficial in preventing perovskite phase segregation or improving the interface. For example, the introduction of TOAB in PSCs has been shown to reduce the PbI_2_ formation and increase the PV performance.^23^

In this study, we performed a facile strategy of postdeposition Au NR treatment of a triple cation ultrathin semitransparent perovskite film. We demonstrate enhancement in the open-circuit voltage and fill factor (FF) due to the plasmonic effect, which results in more than 13% efficiency having 27% AVT. The Au NRs were fabricated and coated with TOAB ligands, which are favorable strategies for both protecting the Au NRs and improving the PSC stability. It reveals that the Au NRs contribute to the enhancement of the carrier transfer and transport at the perovskite/HTM interface. Moreover, impedance spectroscopy shows less trap density and high recombination resistance at this interface due to the plasmonic Au NR treatment.

## Experimental Section

### Au NR Synthesis

The chemicals for this synthesis are purchased from Sigma-Aldrich with 99% purity. Au seeds were synthesized in a water bath at 27 °C by adding 5 μL of 50 mM HAuCl_4_ solution to 0.94 mL of CTAB 0.1 M. The solution was stirred for 5 min; then, 60 μL of freshly prepared NaBH_4_ 10 mM solution was rapidly injected under vigorous stirring. To synthesize the Au NRs, 50 μL of HAuCl_4_ (50 mM) was mixed with 5 mL of CTAB (100 mM) and then kept for 10 min at 27 °C in a water bath. Afterward, 37.5 μL of ascorbic acid solution (100 mM) was added, and the solution was gently shaken for a few seconds until the solution turned colorless. Then, 40 μL of AgNO_3_ (5 mM) was added to the solution. Finally, the seed solution (60 μL) was added, and the solution was vigorously shaken and then left undisturbed at 27 °C for 30 min in a water bath. The solution was centrifuged at 10,000 rpm for 10 min, and the supernatant was taken out. Following that, the ligand exchange process takes place by having 3 mL of Au NR solution mixed with 3 mL of thiomalic acid (10 mM). The pH was adjusted to 9 while stirring. Afterward, 1.5 mL of TOAB in chlorobenzene (50 mM) was added. The resulting mixture was stirred for 30 min until the water phase and organic phase colors switch. The mixture was then washed by centrifugation at 10,000 rpm for 8 min, and the supernatant was taken out. The final concentration of Au NRs is around 0.02 mg/mL.

### Device Fabrication

The organic cations were purchased from GreatCell solar; the lead compounds were purchased from TCI; CsI, SnCl_2_, and spiro chemicals with 99.9% purity were purchased from Sigma-Aldrich. FTO glass was washed with deacon soap, deionized water, and ethanol for 15 min and then subjected to UV–ozone treatment for 15 min. A thin planar layer of SnO_2_ was deposited by spin coating of SnCl_2_·2H_2_O (0.1 M) in ethanol at 1500 rpm for 10 s followed by fast spinning at 5000 rpm for 10 s. The substrates were then annealed at 180 °C for 1 h. After cooling at room temperature, the SnO_2_ substrates were subjected to another UV–ozone treatment for 15 min before being transferred into a nitrogen glove box. Perovskite solution was prepared using 0.6 M PbI_2_, PbBr_2_, CsI, MAI, and FAI in dimethylformamide (DMF) and dimethyl sulfoxide (DMSO) with stoichiometry of Cs_0.05_(MA_0.17_FA_0.83_)_0.95_Pb(I_0.83_Br_0.17_)_3_, and the DMF: DMSO ratio was 4:1. A total of 30–40 μL of perovskite solution was spin-coated on SnO_2_ substrates at 2000 rpm for 10 s followed by 6000 rpm for 30 s. A total of 100 μL of chlorobenzene was dripped in 5 s before spin ends. Substrates were then annealed at 100 °C for 45 min. The Au NR solution is dynamically spin-coated on perovskite at 4000 rpm for 20 min, and 70 mg of spiro-OMeTAD, 28 mL of 4-*tert*-butylpyridine, 16.94 mL of bis(tri-fluoromethylsulfonyl)amine lithium salt, and 35 mL FK209 Co(III) TFSI salt (18.8 mg/50 mL in acetonitrile) were dissolved in 1 mL of chlorobenzene. In total, 40 μL of the solution was spin-coated onto the substrate at 4000 rpm for 30 s. Finally, a 80 nm layer of Au was thermally evaporated at a rate of 0.1–0.2 A/s at an initial stage and increased to 1 A/s at a later stage.

### Characterizations

Scanning electron microscopy (SEM) and transmitted electron detection (TED) images were obtained using a field emission scanning electron microscope, JEOL JSM-7600F, and ZEISS field emission scanning electron microscope. The optical absorption measurement was performed on a UV-3600 (Shimadzu) spectrophotometer. Thin-film X-ray diffraction (XRD) data were collected using a Bruker D8 ADVANCE diffractometer using Cu Kα radiation with a 2θ range of 10 to 60°. The *J*–*V* curves were measured using a SanEI Electric XEC-301S solar simulator under standard simulated AM1.5G illumination at room temperature using a 0.09 cm^2^ mask at 28 mV/s scan rate. Calibration was done using a standard reference silicon cell (Newport). The *J*–*V* measurement was performed after several scans until the results are stabilized. The incident photon-to-current conversion efficiency was measured using a PVE300 (Bentham) with a dual xenon/quartz halogen light source in the direct current mode with a mechanical optical chopper at 30 Hz. The impedance and capacitance–voltage measurements were performed using an electrochemical workstation (AUTOLAB TYPE II). The impedance frequency is done from 1 MHz to 0.1 Hz. Steady-state photoluminescence (PL) was done on a Fluorolog, FL-1057, Horiba Instruments, equipped with a 450 W Xe Lamp with an excitation wavelength of 520 nm. Time-resolved PL (TRPL) dynamics were collected using a micro-PL setup, employing a Nikon microscope, and using a Picoquant PicoHarp 300 time-correlated single-photon counting (TCSPC) system. A picosecond pulsed laser diode, Picoquant P–C–405B, λ = 405 nm with 2.5 MHz repetition (40 Hz frequency divided by a 16 factor) rate, was used as the excitation source. The excitation fluence was <2 μJ/cm^2^. The emitted fluorescence signal was coupled to an avalanche diode synchronized with the excitation laser via TCSPC electronics. The sample for the PL study was glass perovskite with and without Au NRs.

## Results and Discussion

**Figure 1:**
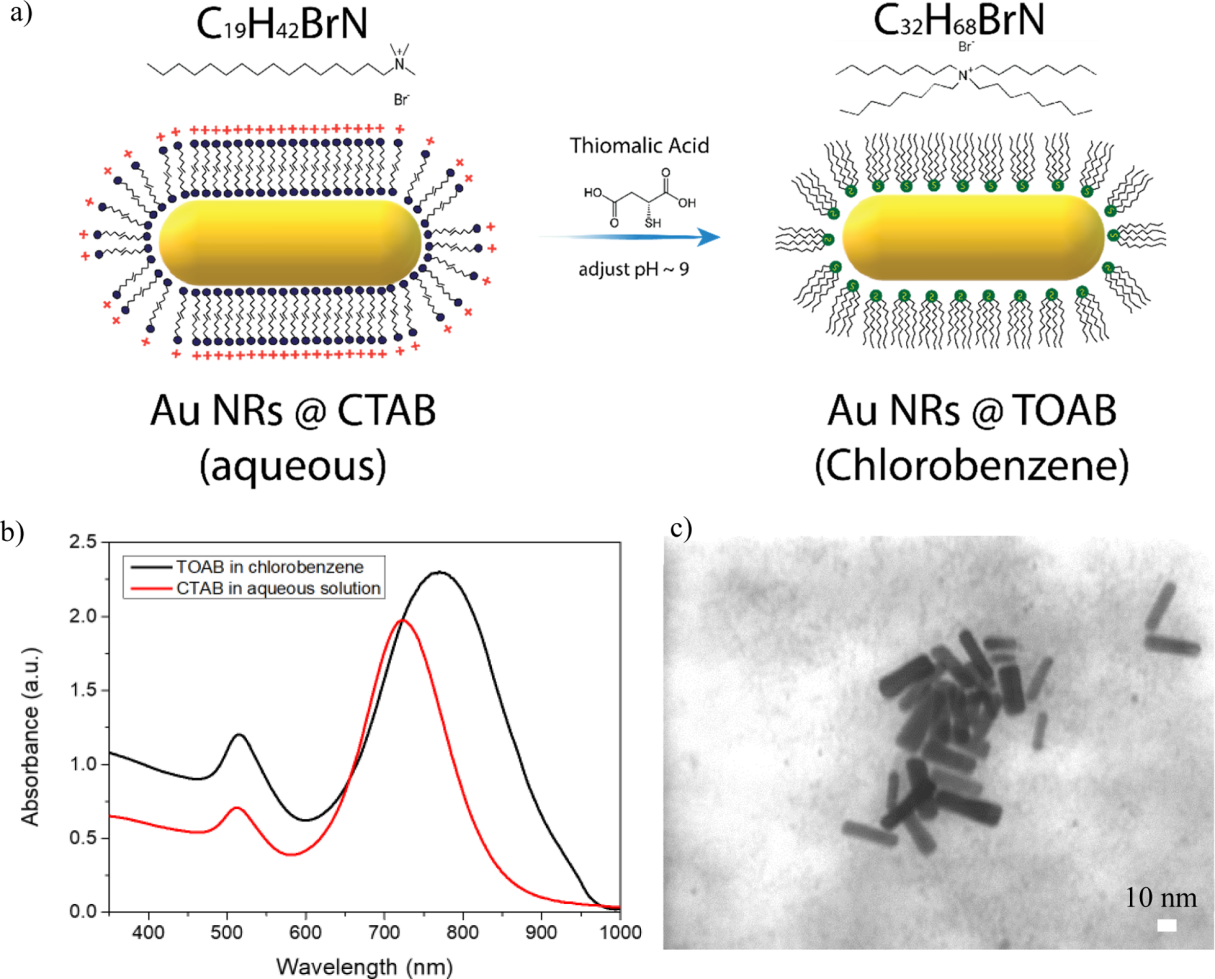
(a) Schematic illustration of the ligand exchange from Au NRs@CTAB to Au NRs@TOAB. (b) Absorbance spectra of Au NRs in aqueous solution and in chlorobenzene solution. (c) SEM–TED image of the Au NRs in chlorobenzene.

The Au NR synthesis was conducted based on the seed-mediated method^20^ in an aqueous solution using CTAB as the ligands. In order to use the Au NRs on top of the perovskite film, it is essential to transfer them to a suitable organic solvent that does not harm the perovskite. Therefore, the Au NRs were transferred to organic medium (i.e., chlorobenzene solvent) using TOAB, which functions as the ligand.^24^Figure 1a shows a schematic illustration of the ligand exchange process. The introduction of thiomalic acid with a thiol functional group can be strongly bonded to the Au NR surface. During continuous stirring, the TOAB exchanges the CTAB ligands, which are attached to the surface of the Au NRs, and stabilize them in the low-polarity solvent chlorobenzene.

Figure 1b shows the absorption spectra of the Au NR solution before and after the ligand exchange. The as-synthesized Au NRs exhibit two resonance peaks at around 510 and 720 nm. The two absorption peaks at higher wavelength belong to the transverse dipole plasmon mode, and the strong NIR peak comes from the longitudinal dipole plasmon mode.^25^ There is a shift and widening in the resonance peak after the ligands exchange to organic medium. The transverse dipoles at 510 nm are the same under both conditions, while the longitudinal dipole plasmon mode shows peak shifts to longer wavelength after the ligand exchange. The full width half maximum increases upon the ligand exchange, which indicates a wider size distribution of the NRs in the organic solution, which might be due to partial agglomeration during the ligand exchange process. Figure 1c shows SEM–TED images of the Au NRs in the final solution before deposition. In terms of size, the synthesized Au NRs have an average length of ca. 35 ± 5 nm and a diameter of ca. 11 ± 2 nm. An additional step was added to improve the Au NR size distribution in order to reduce the number of excess ligands in the solution (Figure S1).

**Figure 2:**
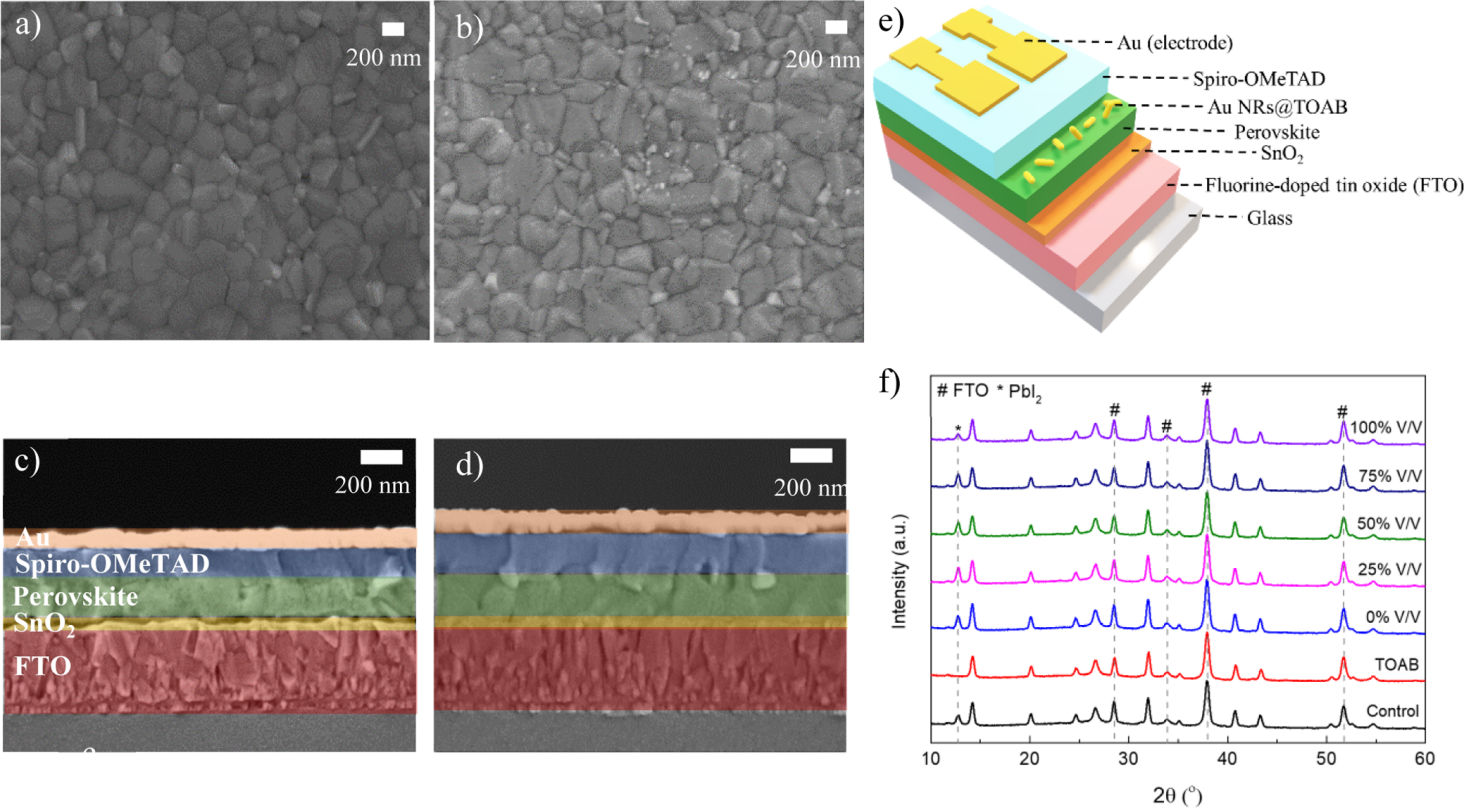
Top-view and cross-sectional SEM image of the perovskite without Au NR treatment (a,c) and perovskite with Au NR treatment (b,d). (e) Schematic illustration of the PSC structure. (f) XRD pattern of the perovskite film as a function of Au NR treatment. V/V indicates the dilution amount where 100% V/V means no dilution.

To investigate the effect of the Au NR postdeposition treatment on the perovskite morphology, both top-view and cross-sectional SEM images were taken with and without Au NR treatment (Figure 2a–d). There are no significant changes in the grain size of the perovskite after the Au NR treatment. The bright nanoparticles shown in Figure 2b indicate the presence of Au NRs on top of the perovskite surface. Chlorobenzene treatment also exhibits no significant change in the morphology of the perovskite film (Figure S2b), while TOAB treatment seems to reduce the grain size of the perovskite (Figure S2a). The unattached TOAB might be detrimental to the grain size as it may attach to Pb and disrupt the crystal structure.^26^ In addition, the amount of TOAB in Au NRs is much less due to the cleaning process. A schematic illustration of the planar PSC used in this study is shown in Figure 2e and consists of the following layers: FTO/SnO_2_/perovskite/spiro-OMeTAD/Au. The cross-sectional SEM image of the completed devices can be seen in Figure 2c,d without and with Au NR treatment, respectively. The thickness of the semitransparent perovskite film is around ∼200 nm, thinner than the typical high-efficiency perovskite’s thickness^27^ which is around 500–700 nm. Moreover, it can be observed that the addition of Au NRs does not affect the perovskite film thickness, while the thickness of the spiro-OMeTAD layer is increased by 30–40 nm as compared to its original thickness. The addition of Au NRs may improve the perovskite surface affinity to spiro-OMeTAD. The change in spiro-OMeTAD thickness can affect the device performance;^28^ however, the change in thickness in this work should not affect the PV performance significantly as was reported previously.^29^

The effect of various amounts of Au NR treatment on the perovskite crystal structure was also investigated by XRD (Figure 2f). The main peaks of the perovskite phase at 14.2, 26.7, 32, and 41° are observed in all the samples.^30^ The absence of shifts in these peaks indicates that the Au NR treatment does not affect the crystallographic structure of the perovskite. In addition, interestingly, at a high amount of Au NR treatment (100% V/V), the amount of PbI_2_ is reduced as indicated by the reduction of the PbI_2_ peak intensity around 12.7°. This phenomenon can be attributed to the introduction of TOAB, which acts as ligands and has been reported to assist in the conversion of PbI_2_ into MAPbI_3_ in a two-step deposition method of perovskite.^23^ It is shown in the XRD that the introduction of only TOAB suppresses the PbI_2_ formation on the perovskite surface (Figure 2f). Moreover, the bromide from the TOAB ligands also assists in improving the stability and reducing the phase segregation of the film as a previous study reported.^31^ On the other hand, excess of TOAB might be detrimental as seen in the poorer grains and by the possible change of the band gap of the fabricated perovskite.^32^

**Figure 3:**
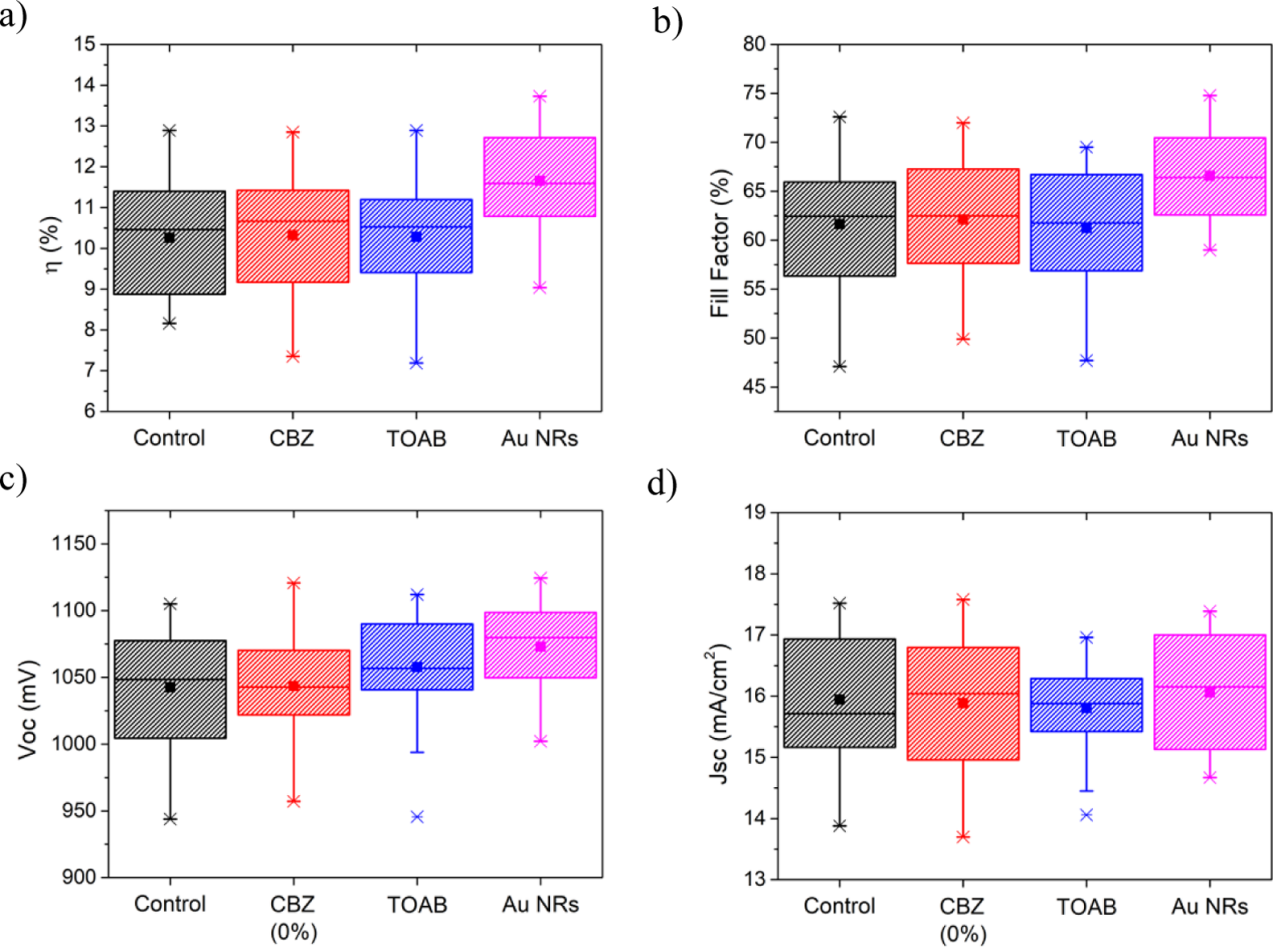
(a–d) PV parameters for different deposition treatments (measured in the reverse-bias scanning mode); control (w/o treatment), CBZ: Chlorobenzene, TOAB: only ligands. 25 samples are measured for each condition. for each Box plots representing data within 25–75 percentile, middle line as the median, square as the mean, and whisker as the outlier.

Figure 3 shows the statistics of the PV parameters of the semitransparent PSCs as a function of various treatments of the perovskite film surface. The PV results show that the improvement is observed in the case of the Au NRs post-treatment, while the solvent (CBZ) or TOAB treatments do not affect the PV performance. The optimal concentration of Au NR solution treatment was studied as presented in Figures S3 and S4 for reverse and forward *JV* scan, respectively. The optimum efficiency was observed at 50% V/V (equivalent to 0.01 mg/mL Au NR solution), and the improvement is mainly due to an increase in the *V*_oc_ and FF, while the *J*_sc_ only increase slightly. The reason behind this efficiency improvement is further investigated and discussed below.

**Figure 4:**
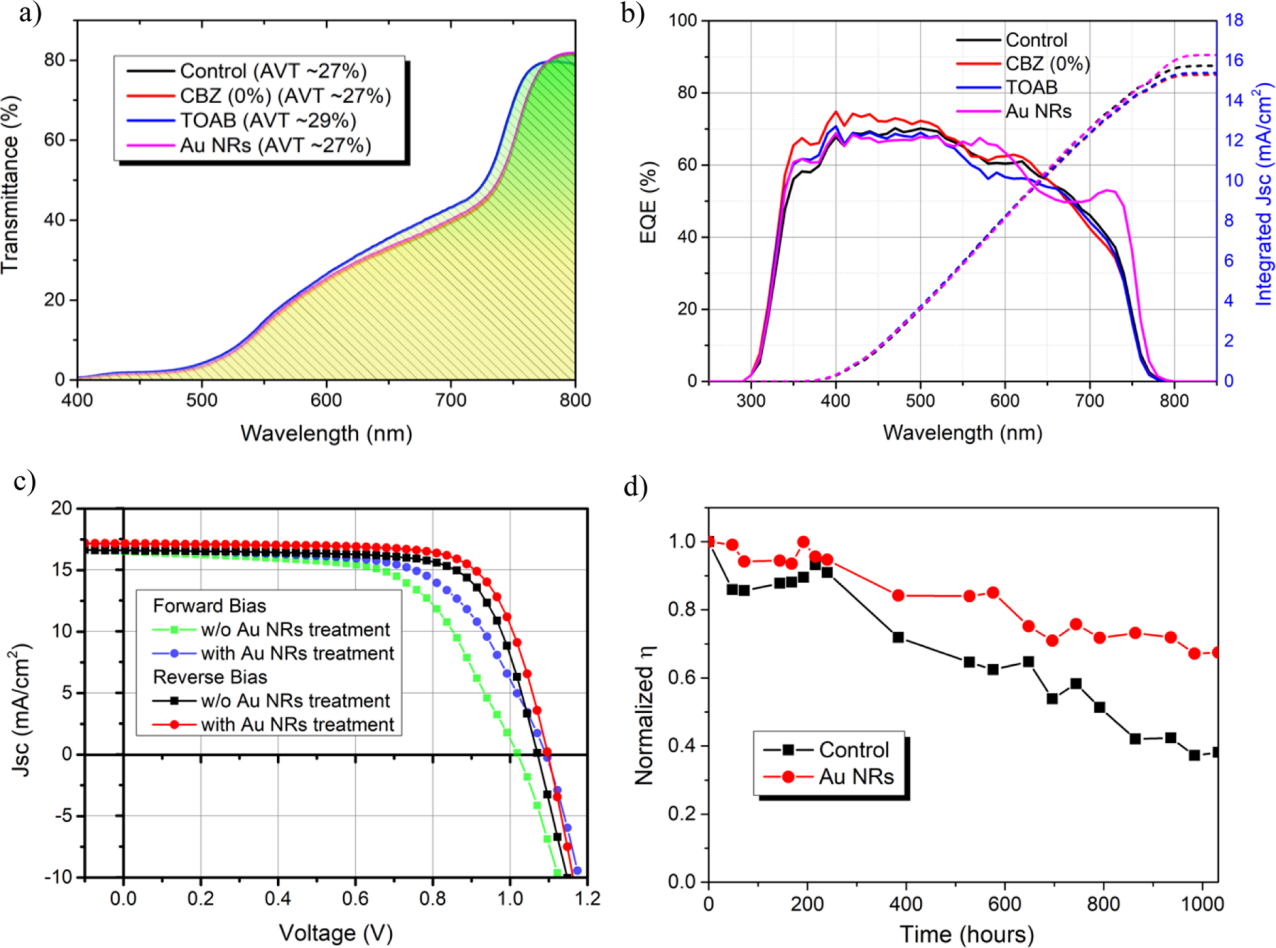
(a) Average transmittance spectra of the perovskite devices (without the Au electrode). The average visible transmittance (AVT) is calculated from average transmittance from 400 to 800 nm as shown by the area under the curve. (b) EQE of the semitransparent perovskite devices including their integrated *J*_sc_. (c) *J*–*V* curve of the champion solar cell for both the control and Au NR-treated PSCs in forward and reverse scans. (d) Stability test for the solar cell with and without the Au NR treatment. Measurements were carried out at ≈80% humidity at room temperature (25 °C) under a reverse-bias scan. After measurements, the devices without encapsulation were stored in a dry box with 20–30% humidity at room temperature.

The transmittance values of these semitransparent PSCs are shown in Figure 4a (see Figure S5 for absorbance spectra). All cases exhibit similar AVT around 27%, which proves the semitransparency properties of the device even after the Au NR treatment (for comparison, high-efficiency nontransparent PSC has an AVT of 2–4%). The slight increase in AVT in the case of TOAB is related mainly to the smaller grains and additional pinholes in the film (Figure S2). Further investigation of the external quantum efficiency (EQE) of these semitransparent PSCs shows evidence of the better charge collection profile for the Au NR-treated PSC in comparison with the control PSC as shown by the sharper band edge (Figure 4b). The sharper edge is translated into a higher FF and *V*_oc_ of the device. In addition, there is also a clear enhancement in the EQE at the long wavelength (low energy) around the longitudinal resonance of the Au NRs. It implies that light at this wavelength was trapped and amplified by the Au NRs and, as a result, absorbed by the perovskite. Therefore, the highest integrated *J*_sc_ is observed for the Au NR-treated sample. The integrated *J*_sc_ from the EQE is in good agreement with the *J*_sc_ measured using the solar simulator. Although the motivation of Au NR incorporation is to improve light absorption, the *J*_sc_ does not improve significantly after the treatment compared with the control. It might be due to nonoptimal distribution and distancing of Au NRs. The distribution of Au NRs affects the plasmonic properties, in particular the field enhancement and light scattering capability.^7^ For example, a study found out that optimal gaps are required for two Au NPs to show absorption enhancement compared with single Au NPs, and with a closer gap, it is detrimental.^33^ In addition, the band gap of perovskite is calculated using the first derivative of the EQE spectra (dEQE/d*E*, where *E* = *hc*/λ),^34^ while no significant changes are observed in all the samples (*E*_g_ = 1.64–1.65 eV).

Figure 4c shows the current–voltage (*JV*) curve of the best-performing PSCs without and with Au NRs. Their PV parameters and diode parameters are tabulated in Table 1. The diode model parameters, that is, ideality factor η_1_, reverse saturation current *J*_01_, series resistance (*R*_s_), and shunt conductance (*G*_sh_), are extracted from their respective dark *JV* curve (Figure S6) using Lambert-W curve fitting method.^35^ In both forward- and reverse-bias scans, Au NR PSCs show higher *V*_oc_, *J*_sc_, and FF compared to the control PSC. This is also accompanied by lower η_1_ and *J*_01_, which determine the quality of the solar cell. In addition, less hysteresis can be observed with the addition of Au NRs at the perovskite/HTM interface. As hysteresis is related to the ionic charge movement and charge accumulation in the perovskite and at the selective contacts,^36^ the Au NR treatment improves the perovskite/HTM interface by passivating defects at this interface, which results in better charge transport and reduction of charge accumulation. The results of this study is comparable with that of the previously reported semitransparent perovskite devices (see Table S2).

**Table 1:** Diode Model *J*–*V* Parameters for the Control and Au NR-Treated PSCs^a^

devices	efficiency (%)	*J*_sc_ (mA/cm^2^)	*V*_oc_ (mV)	FF (%)	η_1_	*J*_01_ (A/cm^2^)	*R*_s_ (Ohm·cm^2^)	*G*_sh_ (mS/cm^2^)
Forward Scan								
control	10.2	16.46	1025.5	60.74	3.53	3.02 × 10–8	2.62	3.00
Au NRs	11.4	16.80	1094.1	62.12	2.45	4.57 × 10–11	5.00	0.15
Reverse Scan								
control	12.9	16.57	1070.8	72.95	1.74	9.40 × 10–14	22.89	0.18
Au NRs	13.7	17.11	1097.1	73.12	1.30	2.60 × 10–17	25.54	0.19

a The diode parameters are extracted by the Lambert-W model on their respective dark IV.

Stability tests of these semitransparent PSCs were conducted under ambient conditions (∼80% humidity at ∼25 °C room temperature). The semitransparent PSCs were unencapsulated and stored in a dry box with 20–30% humidity in the dark. The control PSC starts to decay faster around 50 h and dramatically decay further above 250 h, while the semitransparent PSCs with Au NR treatment show gradual decay with time as shown in Figure 4d. The improved stability of Au NR PSC could be attributed to the positive effect of the TOAB ligands, which passivate the surface of the perovskite by reducing the formation of the PbI_2_ phase, which retards the phase degradation and segregation of the perovskite as observed by XRD. Similar findings have been reported for several organic molecules with “Lewis base” functional groups, for example, thiophene, pyridine, mercaptans, or carbonyl groups.^37^ These molecules assist in the passivation of Pb^2+^ on the perovskite surface due to the electrons’ lone pair. In addition to that, a passivation layer can be formed by alkyl ammonium molecules, which also demonstrates better stability.^38^

**Figure 5:**
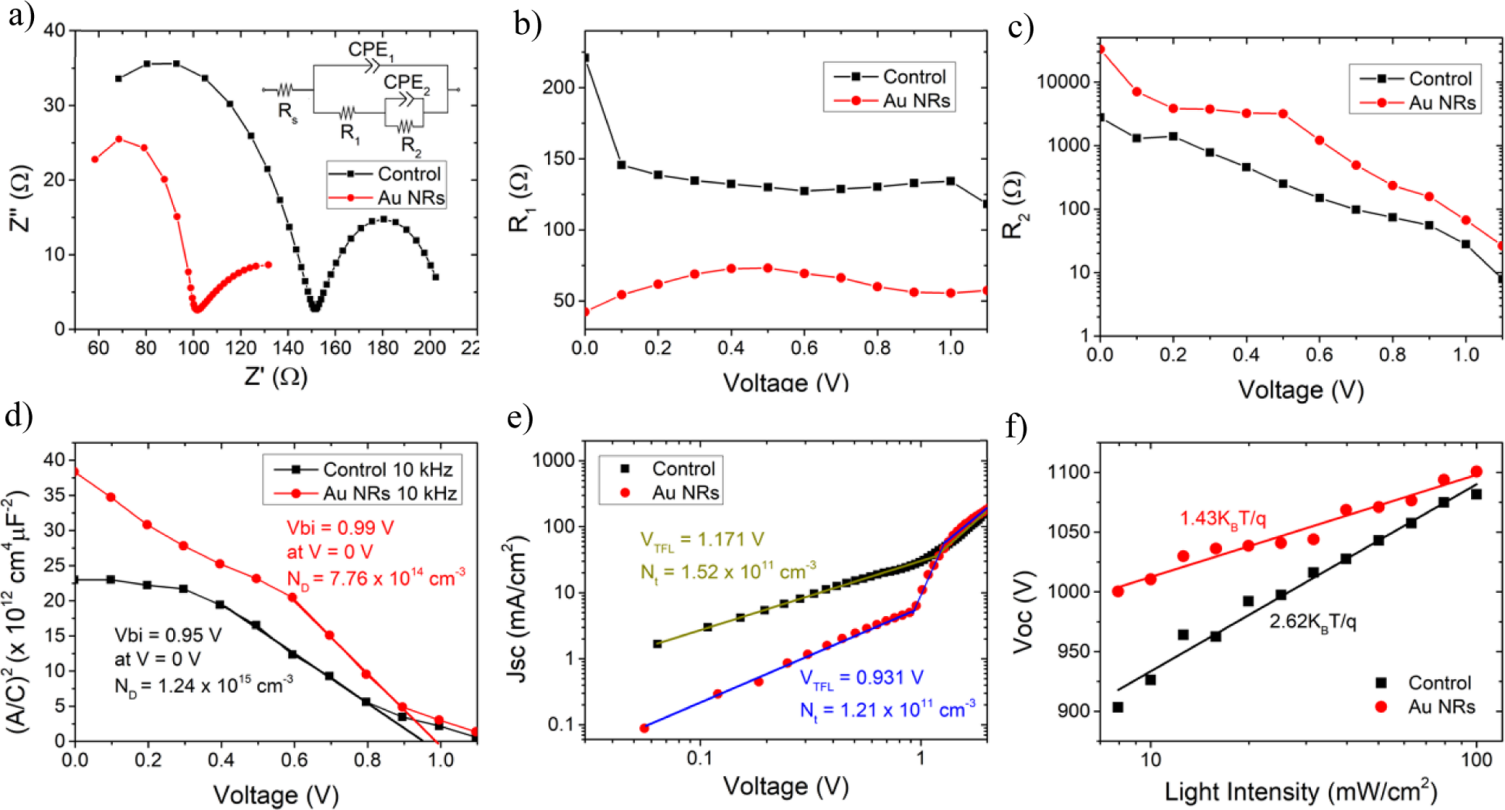
(a) EIS of perovskite cells with and without Au NR treatment under 1 sun illumination at *V*_oc_ bias. Extracted from the EIS: (b) *R*_1_ and (c) *R*_2_ from the equivalent circuit at various bias potentials. (d) Mott–Schottky plots performed at 10 kHz (dark conditions) with ε = 46.9.^39^ (e) *J*–*V* curve from the space-charge limited current (SCLC) device. In this measurement, the perovskite is sandwiched between two HTMs. (f) *V*_oc_ versus light intensity of the perovskite cells.

To further elucidate the effect of the Au NR treatment, several advanced electrical characterizations were performed. Figure 5a exhibits the Nyquist plots from the electrochemical impedance spectroscopy (EIS) spectra of the PSC with and without the Au NR treatment. The measurements were conducted under light illumination at *V*_oc_ bias. The data were fitted and analyzed based on the two-component matryoshka (nested, ladder) equivalent circuit model.^40^ In the Nyquist plot, the high- and low-frequency components are assigned to the signature of the charge transfer resistance (*R*_1_) and the recombination resistance (*R*_2_), respectively.^41^*R*_s_ is the series resistance, which is related to external wiring or cables and contact electrode resistance.^42^Figure 5a shows that Au NR PSCs possess a smaller arc at high frequency but a larger arc at low frequency, which indicates smaller charge transfer resistance and larger recombination resistance compared to those of the nontreated PSCs. *R*_1_ and *R*_2_ of the Au NRs are 55.45 and 66.89 Ω, respectively, compared with those of the control samples, which are 134.28 and 27.89 Ω, respectively, which indicates that Au NR treatment assists in preventing charge recombination at the interface and is beneficial for electron extraction. The EIS measurements were also conducted at scanning potential values from 0 to 1.1 V. *R*_1_ remains smaller for the Au NR-treated PSC for the whole potential range (Figure 5b), which shows better charge transfer resistance and thus a higher FF.^43^ As for the recombination resistance, the value of *R*_2_ (Figure 5c) is larger in the case of the Au NR-treated PSC (lower recombination resistance) for the whole potential range than for the nontreated PSC. At high voltage, the dominant mechanism is charge recombination, while at low voltage, charge transfer dominates.^44^ This explains the difference in these two PSCs’ conditions, especially the higher *V*_oc_ value of the Au NR-treated PSC.

The defect density (*N*_D_) of the absorber and the built-in potential (*V*_bi_) in these PSCs were studied using capacitance–voltage (*C*–*V*) Mott–Schottky analysis (Figure 5d). The measurements were conducted under dark conditions with potential bias at 10 kHz from 0 to 1.1 V. The built-in potential (*V*_bi_) extracted from the measurement is 0.99 V for the Au NR-treated PSC and 0.95 V for the nontreated PSC. *V*_bi_ is closely related to the *V*_oc_ of the solar cell; in the case of the Au NR-treated PSC, *V*_bi_ is higher and closer to *V*_oc_, which is in line with the PV performance of the PSC.

Furthermore, the acceptor density can be calculated using the equation , where *C* is capacitance, *q* is the electron charge, *A* is the area, ε_r_ is the dielectric constant of the absorber, ε_0_ is the dielectric constant of free space, *N*_D_ is the defect density, *V* is the potential bias, and *V*_bi_ is the built-in potential.^45^ ε_r_ in this study is 46.9, which is based on the dielectric constant of FAPbI_3._ This value is widely invariant from triple cation perovskite according to previous reports.^39^ The *N*_D_ values at *V* = 0 are calculated to be 7.78 × 10^14^ and 1.24 × 10^15^ cm^–3^ for Au NRs and control PSCs, respectively. Based on that, it was observed that the Au NR-treated PSCs have lower defect density than that of the nontreated PSCs at different applied biases.

To further probe the effect of Au NRs on the defect density, a space-charge limiting current (SCLC) analysis was conducted. In this experiment, PSCs, where the perovskite is sandwiched between hole transport materials (FTO/NiO/perovskite/Spiro-OMeTAD/Au), were fabricated. By measuring the dark current of these devices (Figure 5e), the trap density can be extracted using the equation , where is ε_r_ is the dielectric constant of the absorber, ε_0_ is the dielectric constant of free space, *q* is the elementary charge, *L* is the thickness of the measured material, and *V*_TFL_ is the voltage at the trap-filled limited (TFL) regime, which occurs when there is a change in the *JV* response from the Ohmic behavior indicated by the change of the *JV* curve slope.^46^*V*_TFL_ indicates the voltage at which the current flow is starting to be limited by the traps in the absorber, which can assist in deducing the number of traps. Based on the extracted parameters from the graph, the hole trap densities of the devices with and without Au NRs are calculated to be 1.21 × 10^11^ and 1.52 × 10^11^ cm^–3^, respectively. A slight reduction in trap density is observed for the Au NRs post-treated PSCs, in good agreement with the Mott–Schottky plot measurements, which confirm the benefits of the Au NRs to the perovskite/HTM interface. The device responses under different light intensities were recorded. *V*_oc_ as a function of the light intensity is plotted in Figure 5f. The control PSC exhibit a slope of 2.62 *k*_B_*T*/*q*, while the Au NR-treated PSC shows a smaller slope of 1.54 *k*_B_*T*/*q*, where kB is the Boltzmann constant, *T* is the temperature, and *q* is the elementary charge. The deviation of the slope from *k*_B_*T*/*q* implies defect-assisted recombination in the devices or ideality factor.^47^ The control sample exhibits a slope of more than 2, which is uncommon for PSCs as the value is usually between 1 and 2. Some studies reported perovskite devices with an ideality factor of more than 2 and correlated this finding with the interface issue.^8^ A theoretical study propose an analytical model, which takes into account ion migration or voltage-dependant issues in PSCs.^9^ It interprets that the ideality factor of more than 2 is related to the electron-limited recombination mechanism, which can be either electron-limited bulk Shockley–Read–Hall (SRH) recombination or perovskite/HTL interface SRH recombination. In our study, the perovskite/HTL interface forms the SRH recombination mechanism since significant changes were observed after the Au NR interface treatment. Therefore, the Au NR-treated PSCs, which show a smaller slope, were able to passivate the interface that leads to lower nonradiative recombination loss and a higher *V*_oc_ than the nontreated PSC. In the case of *J*_sc_ versus light intensity, both samples show a linear relation with no significant difference, as observed in figure S7

**Figure 6:**
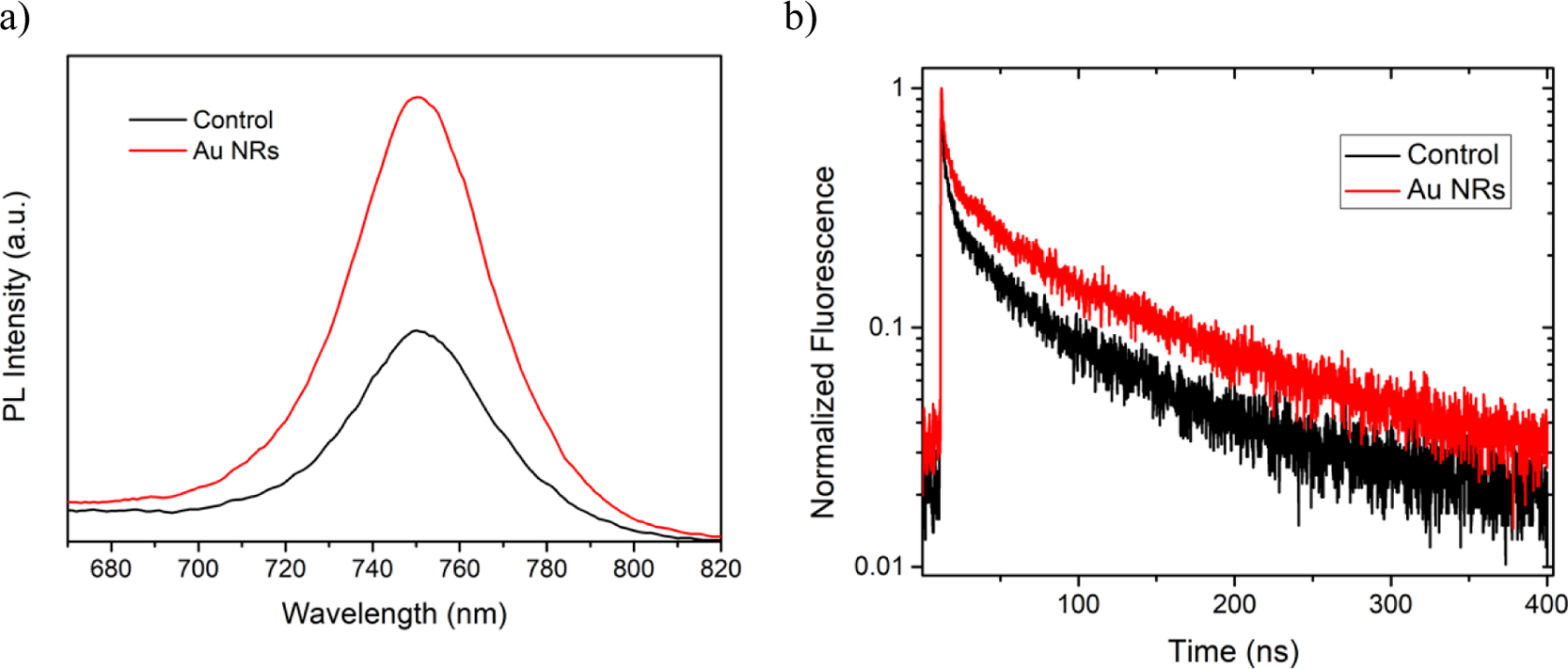
(a) PL spectra and (b) normalized TRPL of the perovskite film with and without the Au NR treatment.

Steady-state PL and TRPL spectra were characterized to understand the effect of the charge recombination process in the perovskite layer for the two cases (Figure 6). The Au NR treatment does not affect the PL peak position, but it enhances the PL intensity of the sample. This observation indicates the suppression of nonradiative recombination in the Au NR post-treated samples. This finding is in agreement with the previous EIS and SCLC measurements, which show lesser trap densities. The higher PL intensity also suggests that the Au NR resonance might amplify the PL of the perovskite. As reported previously, certain metal nanostructures such as nanorods can enhance the fluorescence emission near the band gap, thus increasing the radiative recombination rate and the spontaneous attenuation rate concurrently.^48^ The TRPL values of the Au NRs post-treated and nontreated perovskite films were measured and are reported in Figure 6b. The decays have been fitted with a double exponential decay and are tabulated in Table S1. The faster (τ_1_) characteristic time is correlated to quenching via trap or interfacial charge transfer, and the slower (τ_2_) time is described as free carrier recombination.^49^ Compared to the control sample, both lifetime components of the Au NR post-treated increased; moreover, the addition of Au NRs also increases the fraction of free carrier recombination in the perovskite film. These results are consistent with the previous characterizations.

## Conclusions

In this work, we demonstrate semitransparent PSCs with a PCE of 13.7% and AVT of 27% using a facile Au NR treatment. It was revealed that in addition to the effect of the Au NR plasmons, the TOAB ligands add an extra benefit by reducing the PbI_2_ phase segregation as observed from the XRD measurements. The semitransparent Au NR PSCs show enhancement in the PV performance without compromising the transparency of the cells. An increase in *V*_oc_ and the FF was observed due to better carrier transport and carrier extraction at the interface of the perovskite and the HTM. EIS, SCLC, PL, and Mott–Schottky characterizations demonstrate less trap and defect density, less charge transfer resistance, and less nonradiative recombination for the Au NR-treated PSCs, which result in better PV parameters. Improvement in carrier collection especially near the Au NR resonance peak and the perovskite band gap was observed. The unencapsulated semitransparent Au NR-treated PSCs show enhanced stability over 1000 h.

This promising approach provides a new interface treatment to improve high-efficiency and stable semitransparent PSCs while maintaining their transparency.

## Supporting Information Available

SEM–TED images of Au NR solutions with CTAB and TOAB-unwashed; top-view SEM of the perovskite film with TOAB and chlorobenzene treatments; PV parameters of PSCs (reverse bias scanning mode) with different degrees of Au NR treatment; PV parameters of PSCs (forward bias scanning mode) with different degrees of Au NR treatment; absorbance spectra of perovskite devices (without an Au electrode); dark *J*–*V* curve for the perovskite device with and without the Au NR treatment; *J*_sc_ versus light intensity of the perovskite with and without the Au NR treatment; fitting decay times of the perovskite films with and without the Au NR treatment; and list of notable reported semitransparent perovskite devices with area, AVT, and PCE (PDF)
