# Supplementary material for: Semitransparent Perovskite Solar Cells with > 13% Efficiency and 27% Transperancy Using Plasmonic Au Nanorods
Source: ACS Appl Mater Interfaces. 2022 Feb 24;14(9):11339–49. doi: 10.1021/acsami.1c22748 (PMC8915162; doi:10.1021/acsami.1c22748)
Supplement: Supplementary file 1 — am1c22748_si_001.pdf [file am1c22748_si_001.pdf]

Supporting information

Semitransparent perovskite solar cells with > 13% efficiency and 27% transparency using plasmonic Au Nanorods

Stener Lie[a,b], Annalisa Bruno[c], Lydia Helena Wong[a,b], and Lioz Etgar[a,d,*]

[a]Singapore-HUJ Alliance for Research and Enterprise (SHARE), Nanomaterials for Energy and Energy-Water Nexus (NEW), Campus for Research Excellence and Technological Enterprise (CREATE), Singapore 138602, Singapore;

[b]School of Material Science and Engineering, Nanyang Technological University, Singapore 639798, Singapore;

[c]Energy Research Institute @Nanyang Technological University (ERI@N), Singapore 637141, Singapore;

[d]The Hebrew University of Jerusalem, Institute of Chemistry, Casali Center for Applied Chemistry, Jerusalem 91904, Israel;

*Corresponding author.

E-mail: lioz.etgar@mail.huji.ac.il

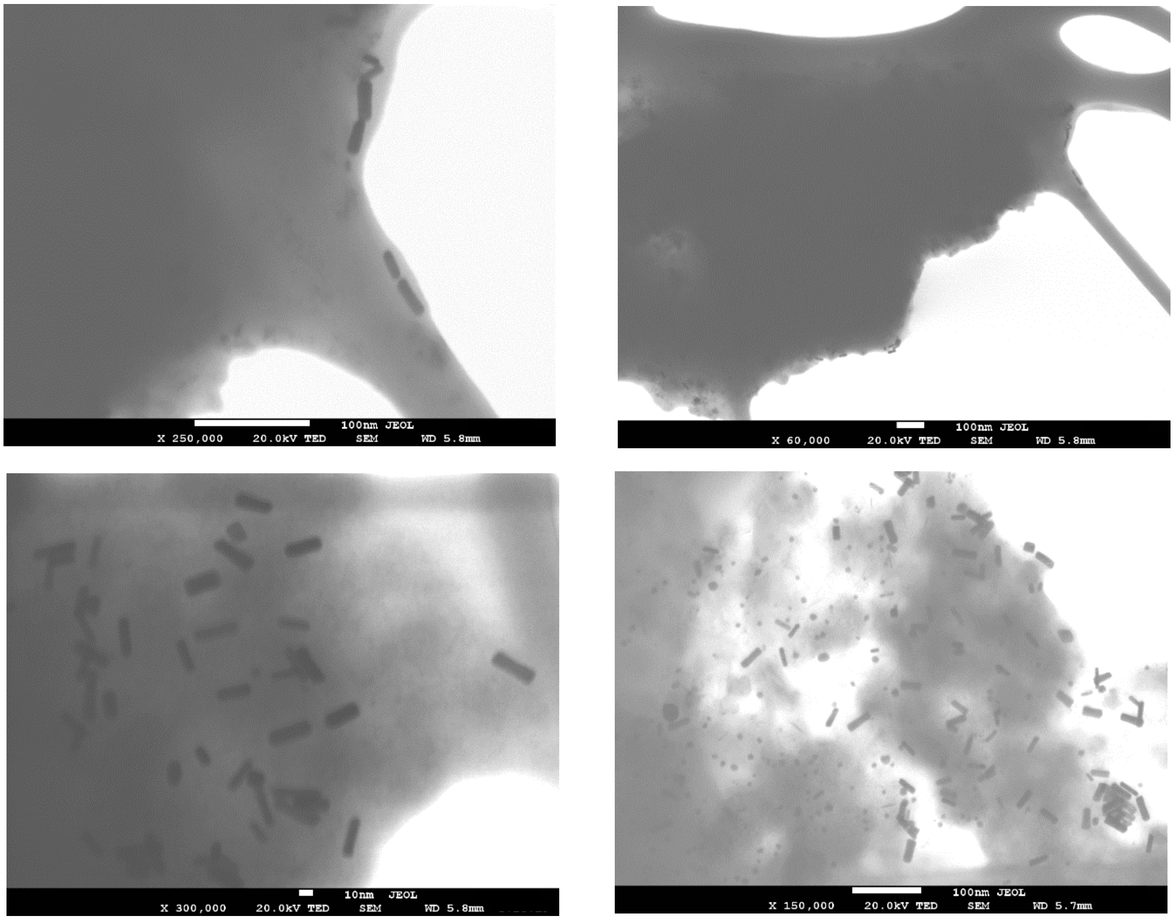

Figure S1. SEM-TED images of Au NRs solutions with CTAB (top) and TOAB-unwashed (bottom)

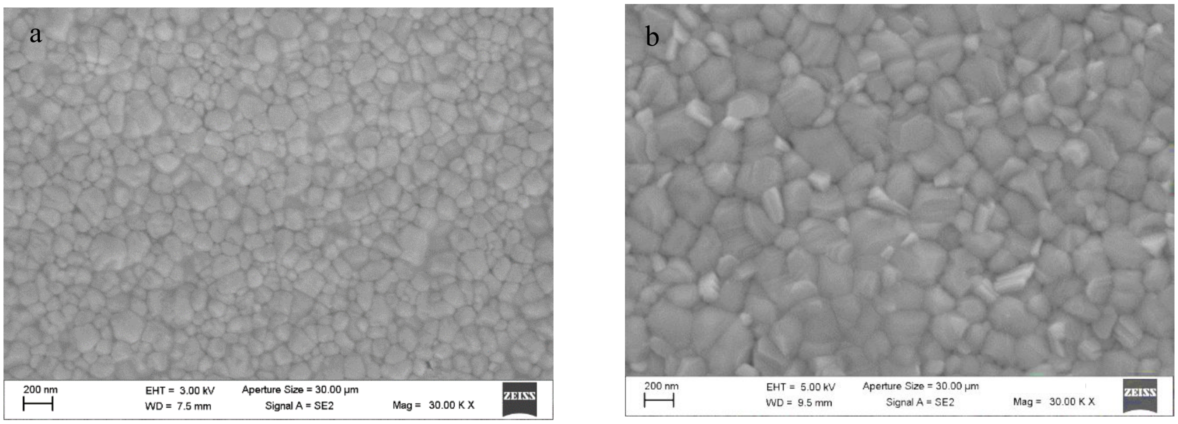

Figure S2. Top view SEM of perovskite film with a) TOAB solution treatment and b) chlorobenzene treatment.

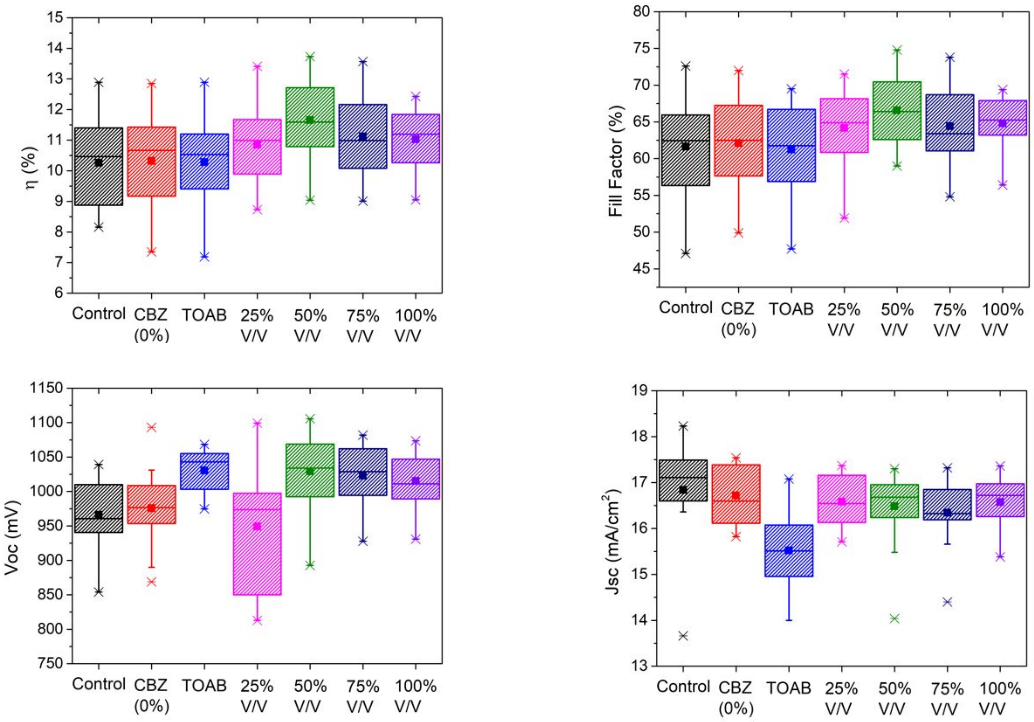

Figure S3. PV parameters of perovskite solar cells with different amount of Au NRs treatment, measured in reverse bias scanning mode.

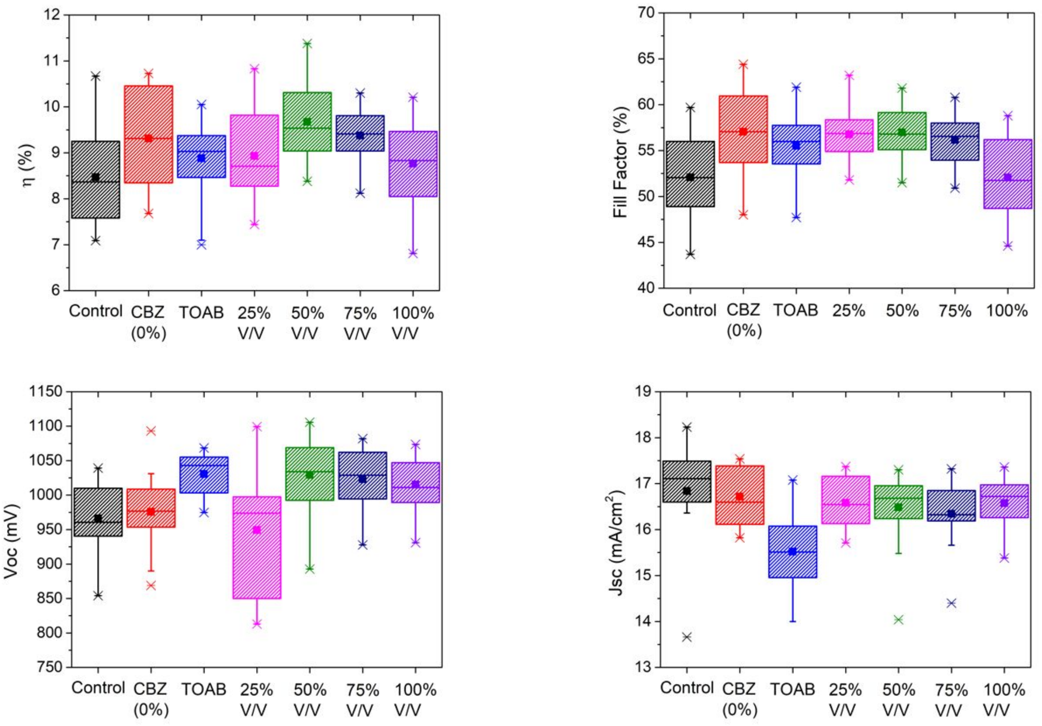

Figure S4. PV parameters of perovskite solar cells with different amount of Au NRs treatment, measured in forward bias scanning mode.

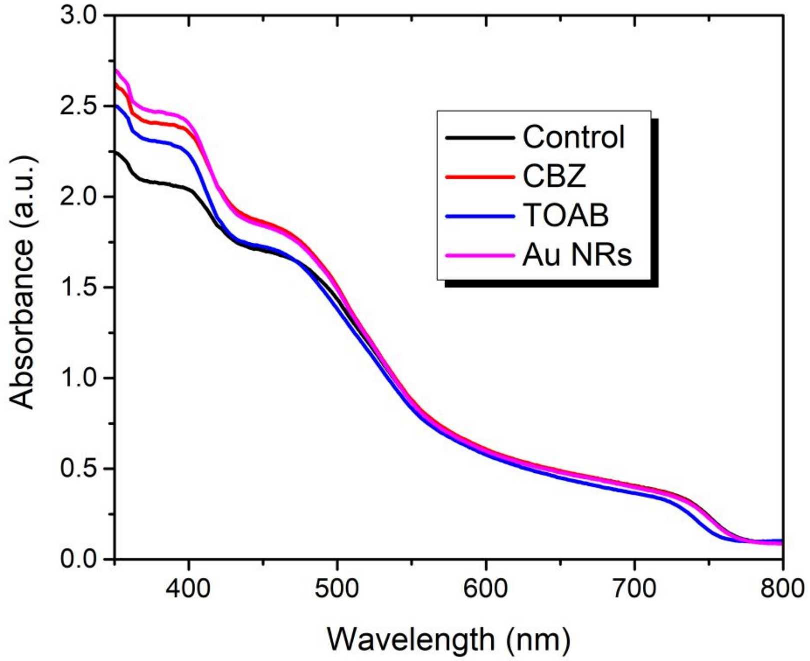

Figure S5. Absorbance spectra of perovskite devices (without Au electrode) from 350 to 800 nm.

a

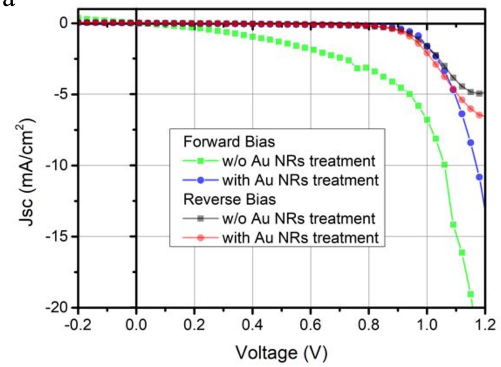

b

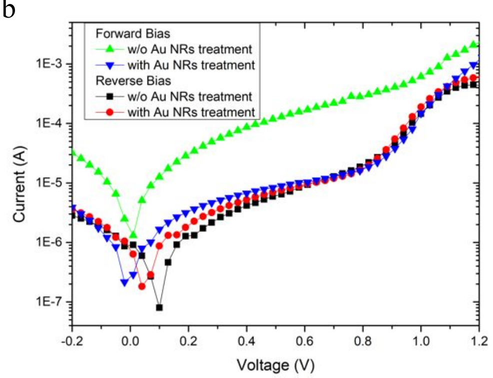

Figure S6. Dark J-V curve for perovskite device with and without Au NRs treatment a) in linear scale and b) logarithmic scale

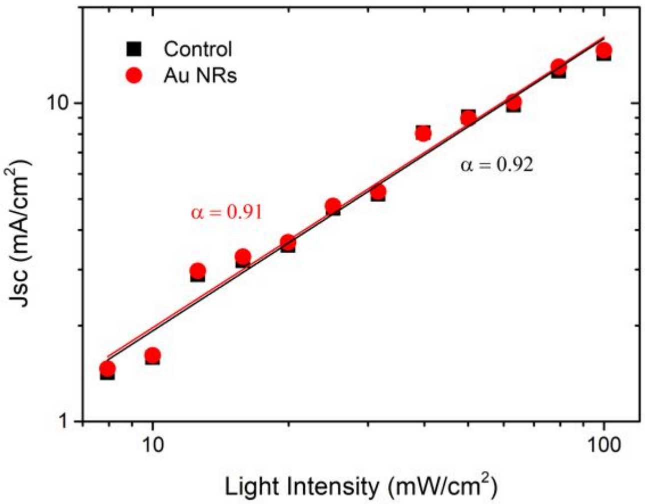

Figure S7. Jsc vs light intensity of perovskite with and without Au NRs treatment

Table S1. Fitting decay times of the perovskite films with and without Au NRs treatment on quartz-glass substrates.

Samples	τ1 (ns)	Fraction 1 (%)	τ2 (ns)	Fraction 2 (%)	τav (ns)
Control	2.455	68.1%	57.69	31.9%	20.06
Au NRs	3.821	59%	82.52	41%	36.11

Table S2. List of notable reported semi-transparent perovskite devices, with area, AVT and PCE. Reproduced with permission from ref. [1] Copyright 2021, John Wiley and Sons.

**Method**	**AVT (%)**	**Area (cm^2)**	**PCE (%)**
Perovskite on Anodized Al_2O_3 Scaffold[2]	33.4	0.06	9.6
Dual Scaffold[3]	23	0.1	8.21
islands-structure-$MAPbI_{3-x}Cl_x$-NiO[4]	27	0.1	10.06
Polarizable thiourea vapor as a 'molecular glue'[5]	24	0.1	9.3
$MAPbI_{3-x}Br_x$ ($0 < x \leq 3$) with Au/MoO_3 contact[6]	21	0.07	9.82
$MAPbI_3$ sandwiched between PEDOT and PCBM, Au/LiF contact[7]	22	0.12	7.3
Nano pillared perovskite on Anodized Al_2O_3 Scaffold[8]	30	40.8	9.04
Two-step linear facing target sputtering[9]	24.7	1.08	13.61
Solution-Processed Thiol-Functionalized Surfactant as Buffer Layer[10]	20.8	0.12	11.8
ALD to deposit ZnO and Al_2O_3 films as cathode buffer layer[11]	25.5	0.04	10.8
Thin triple cation perovskite with Europium doping and employing a down-converting phosphor material[1]	20.3	21	9.5

(1) Rai, M.; Yuan, Z.; Sadhu, A.; Leow, S. W.; Etgar, L.; Magdassi, S.; Wong, L. H. Multimodal Approach Towards Large Area Fully Semitransparent Perovskite Solar Module. *Advanced Energy Materials* **2021**, *11* (45). DOI: 10.1002/aenm.202102276.
(2) Kwon, H. C.; Kim, A.; Lee, H.; Lee, D.; Jeong, S.; Moon, J. Parallelized Nanopillar Perovskites for Semitransparent Solar Cells Using an Anodized Aluminum Oxide Scaffold. *Advanced Energy Materials* **2016**, *6* (20), 1601055.
(3) Xiao, S.; Chen, H.; Jiang, F.; Bai, Y.; Zhu, Z.; Zhang, T.; Zheng, X.; Qian, G.; Hu, C.; Zhou, Y. Hierarchical Dual-Scaffolds Enhance Charge Separation and Collection for High Efficiency Semitransparent Perovskite Solar Cells. *Advanced Materials Interfaces* **2016**, *3* (17), 1600484.

(4) Wang, Y.; Mahmoudi, T.; Yang, H.-Y.; Bhat, K. S.; Yoo, J.-Y.; Hahn, Y.-B. Fully-Ambient-Processed Mesoscopic Semitransparent Perovskite Solar Cells by Islands-Structure-Mapbi3-Xclx-Nio Composite and Al2o3/Nio Interface Engineering. *Nano Energy* **2018**, *49*, 59-66.
(5) Bag, S.; Durstock, M. F. Efficient Semi-Transparent Planar Perovskite Solar Cells Using a 'Molecular Glue'. *Nano Energy* **2016**, *30*, 542-548.
(6) Yuan, L.; Wang, Z.; Duan, R.; Huang, P.; Zhang, K.; Chen, Q.; Allam, N. K.; Zhou, Y.; Song, B.; Li, Y. Semi-Transparent Perovskite Solar Cells: Unveiling the Trade-Off between Transparency and Efficiency. *Journal of Materials Chemistry A* **2018**, *6* (40), 19696-19702.
(7) Roldan-Carmona, C.; Malinkiewicz, O.; Betancur, R.; Longo, G.; Momblona, C.; Jaramillo, F.; Camacho, L.; Bolink, H. J. High Efficiency Single-Junction Semitransparent Perovskite Solar Cells. *Energy & Environmental Science* **2014**, *7* (9), 2968-2973.
(8) Kwon, H.-C.; Ma, S.; Yun, S.-C.; Jang, G.; Yang, H.; Moon, J. A Nanopillar-Structured Perovskite-Based Efficient Semitransparent Solar Module for Power-Generating Window Applications. *Journal of Materials Chemistry A* **2020**, *8* (3), 1457-1468.
(9) Lim, S.-H.; Seok, H.-J.; Kwak, M.-J.; Choi, D.-H.; Kim, S.-K.; Kim, D.-H.; Kim, H.-K. Semi-Transparent Perovskite Solar Cells with Bidirectional Transparent Electrodes. *Nano Energy* **2021**, *82*, 105703.
(10) Chang, C.-Y.; Chang, Y.-C.; Huang, W.-K.; Lee, K.-T.; Cho, A.-C.; Hsu, C.-C. Enhanced Performance and Stability of Semitransparent Perovskite Solar Cells Using Solution-Processed Thiol-Functionalized Cationic Surfactant as Cathode Buffer Layer. *Chemistry of Materials* **2015**, *27* (20), 7119-7127.
(11) Chang, C.-Y.; Lee, K.-T.; Huang, W.-K.; Siao, H.-Y.; Chang, Y.-C. High-Performance, Air-Stable, Low-Temperature Processed Semitransparent Perovskite Solar Cells Enabled by Atomic Layer Deposition. *Chemistry of Materials* **2015**, *27* (14), 5122-5130.
